# Editorial: Exploring human-autonomous interactions: agency, awareness, and ethical implications

**DOI:** 10.3389/fpsyg.2026.1843708

**Published:** 2026-05-07

**Authors:** Salvatore Lo Bue, Arthur Prével, Adriana Salatino

**Affiliations:** 1 Department of Life Sciences, Royal Military Academy, Brussels, Belgium; 2 Univ. Lille, CNRS, UMR 9193 – SCALab – Sciences Cognitives et Sciences Affectives, Lille, France

**Keywords:** agency, artificial intelligence (AI), decision-making, human performance, human-autonomous systems interaction, responsibility

## Introduction

Autonomous systems are increasingly woven into the fabric of contemporary life, influencing domains as diverse as mobility, online consumption, public health, defense, and everyday digital interactions (Sutton et al., 2020; Bestyuk and Pokhnatiuk, 2025). As these systems gain autonomy, adaptivity, and decision-making capacity, their influence on human cognition, moral judgment, and responsibility becomes both deeper and more complex. This expanding overlap between human and machine agency raises profound questions about trust, accountability, and the boundaries of human control (Rovira et al., 2007; Prével et al., 2022), questions that require careful examination as autonomous systems become integral to both civilian life and security.

## Research Topic content

The ambition of the present Research Topic is to highlight this depth and complexity. Specifically, we sought contributions examining how human agency is shaped, enhanced, or diminished through interactions with autonomous technologies, and how individuals navigate ethical decisions in high-autonomy environments.

The need for such investigations has never been more urgent. While autonomous systems can increase efficiency, reduce workload, and support more consistent decision-making, they can also lead to diminished situational awareness, skill decay, miscalibrated trust (Endsley, 2017; Haslbeck and Hoermann, 2016), and responsibility shift (Berberian et al., 2012; Salatino et al., 2025a,b,c). This Research Topic brings together four original studies that approach these challenges from complementary disciplinary angles: cognitive science, human–machine teaming, psycholinguistics, and transportation psychology. Collectively, they provide new empirical insight into the cognitive, linguistic, and psychological mechanisms that govern human-autonomous interactions, and they propose conceptual tools to guide future design and regulations.

The contribution by Cao and Scheibehenne investigates how people process skewed outcome distributions when making decisions in environments resembling e-commerce rating systems. By examining how individuals sample information from customer reviews histograms, the study reveals strong biases toward rare, extreme outcomes, despite incentives for accuracy. Beyond confirming traditional cognitive explanations linked to probability weighting, the findings highlight a deeper mechanism: people correct for their biased, samples but only partially.

This work enriches our understanding of human–algorithm interactions by showing how biased sampling can spread through digital choice environments where platforms often highlight surprising or infrequent events. Cao and Scheibehenne's findings suggest that such environments may steer users toward overemphasizing rare signals, even in the presence of corrective cognitive strategies. These insights have implications for political information exposure, trust in automated recommendations, and calibration of human judgments in mixed human–AI systems.

The second article, by van Diggelen et al. addresses a central ethical challenge in human–autonomous teaming for Defense applications: establishing and maintaining meaningful human control (MHC). Using the repertory grid technique, the authors show how experts across disciplines, including military operations, philosophy, engineering, conceptualize MHC. Their findings reveal both agreement among the experts (e.g., the moral sensitivity required of systems and operators) and disagreement (e.g., the nature of autonomy and acceptable levels of system initiative). The study provides a practical framework and shared vocabulary for integrating MHC into military technologies, highlighting that MHC is a multidimensional construct, extending beyond “human in the loop” to include cognitive, moral, organizational, and technical factors.

Language is one of the most subtle yet powerful mediators of human perception, and the study by Petersen and Almor examines how linguistic agentive framing shapes responsibility attribution when “AI” systems cause harm. By manipulating whether an artificial agent is described in agentive (“Dr. AI advised…”) vs. instrumental (“The AI system was used to advise…”) terms, the authors demonstrate that framing significantly alters how people assign responsibility, particularly among individuals having less experience with such systems.

Importantly, the study shows that domain experience moderates this effect: participants familiar with algorithmic systems were less influenced by anthropomorphic or agentive language, suggesting that experience provides a cognitive buffer against unintended anthropomorphism. These findings have implications for public communication surrounding autonomous systems. Agentive descriptions, common in marketing and scientific communication, may unintentionally shift perceived responsibility from developers and organizations to the user and the systems themselves. This highlights the need for careful and transparent language, particularly in high-stakes contexts like health, law, and defense.

The fourth study, by Aasvik et al., extends research on human–autonomous to mobility. Using a large national sample in Norway, the authors examine how informational conditions and individual differences shape acceptance of shared automated vehicles (SAVs). Contrary to expectations, experimentally manipulated features (e.g., steward presence, seating configuration, co-passenger characteristics) did not significantly influence acceptance of SAVs. Instead, stable psychological traits were key elements: openness and agreeableness predicted higher acceptance, while neuroticism and social dominance orientation predicted lower acceptance. Overall, acceptance appears to be driven less by surface-level cues and more by deeper psychological predispositions, highlighting the need for communication strategies that address diverse user profiles.

## Outlook

Taken together, the articles in this Research Topic reinforce a central message: human–autonomy interaction cannot be fully understood through technological capabilities alone. Instead, it emerges from a complex interplay of cognitive biases, moral expectations, linguistic interpretations, personality traits, and context. Three key themes emerge. First, whether through biased sampling, shifting responsibility perceptions, or variable acceptance of automation, human agency can be bolstered or undermined by design choices and communication strategies. Second, establishing meaningful human control, particularly in high-stakes environments such as Defense, demands input from engineers, ethicists, operators, policymakers, and cognitive scientists. Third, psychological and linguistic factors are as influential as technical ones in shaping public trust, responsibility judgments, and willingness to adopt new technologies.

The diversity of approaches showcased in this Research Topic reflects the growing recognition that human–autonomous interaction is not a single field, but a convergence of multiple disciplines. As autonomous systems continue to proliferate, future research will need to explore not only how humans adapt to these systems, but also how systems should be adapted to human cognitive and moral capacities.

We hope that this Research Topic stimulates further inquiry into the boundaries, challenges, and opportunities of human–autonomous collaboration, ultimately contributing to the responsible and ethically grounded development of autonomous technologies.
